# Antibiotic-induced depletion of *Clostridium* species increases the risk of secondary fungal infections in preterm infants

**DOI:** 10.3389/fcimb.2022.981823

**Published:** 2022-08-31

**Authors:** Dabin Huang, Huixian Li, Yuying Lin, Jinting Lin, Chengxi Li, Yashu Kuang, Wei Zhou, Bing Huang, Ping Wang

**Affiliations:** ^1^ Department of Neonatology, Guangzhou Women and Children’s Medical Center, Guangzhou Medical University, Guangzhou, China; ^2^ Department of Data Center, Guangdong Provincial People’s Hospital, Guangzhou, China; ^3^ Department of Pediatrics, Guangzhou Medical University, Guangzhou, China; ^4^ Division of Birth Cohort Study, Guangzhou Women and Children’s Medical Center, Guangzhou Medical University, Guangzhou, China; ^5^ Department of Gastroenterology, Guangdong Provincial Key Laboratory of Gastroenterology, Nanfang Hospital, Southern Medical University, Guangzhou, China

**Keywords:** neonatal fungal infection, antibiotics, intestinal microbiome, *Clostridium* species, SCFA, IL10, IgA

## Abstract

Preterm infants or those with low birth weight are highly susceptible to invasive fungal disease (IFD) and other microbial or viral infection due to immaturity of their immune system. Antibiotics are routinely administered in these vulnerable infants in treatment of sepsis and other infectious diseases, which might cause perturbation of gut microbiome and hence development of IFD. In this study, we compared clinical characteristics of fungal infection after antibiotic treatment in preterm infants. As determined by 16S rRNA sequencing, compared with non-IFD patients with or without antibiotics treatment, *Clostridium* species in the intestinal tracts of patients with IFD were almost completely eliminated, and *Enterococcus* were increased. We established a rat model of IFD by intraperitoneal inoculation of *C. albicans* in rats pretreated with meropenem and vancomycin. After pretreatment with antibiotics, the intestinal microbiomes of rats infected with *C. albicans* were disordered, as characterized by an increase of proinflammatory conditional pathogens and a sharp decrease of *Clostridium* species and *Bacteroides*. Immunofluorescence analysis showed that *C. albicans*-infected rats pretreated with antibiotics were deficient in IgA and IL10, while the number of Pro-inflammatory CD11c^+^ macrophages was increased. In conclusion, excessive use of antibiotics promoted the imbalance of intestinal microbiome, especially sharp decreases of short-chain fatty acids (SCFA)-producing *Clostridium* species, which exacerbated the symptoms of IFD, potentially through decreased mucosal immunomodulatory molecules. Our results suggest that inappropriate use of broad-spectrum antibiotics may promote the colonization of invasive fungi. The results of this study provide new insights into the prevention of IFD in preterm infants.

## Introduction

Invasive fungal disease (IFD) is one of the late-onset severe infections in premature infants (Kilpatrick et al., 2021). Because premature infants and those with low birth weights are at increased risk, neonatal intensive care units (NICU) tend to be locales of relatively high incidences of candidiasis. IFD affects 7 to 10% of preterm infants with birth weights of less than 1500 g (Benjamin et al., 2010; Weimer et al., 2021). Infants with birth weights of less than 750 g are at disproportionate risk: the incidence of IFD in these extremely low birth-weight infants due to infection with *Candida* species is two times higher than in infants with birth weights of 750 to 1000 g (Aliaga et al., 2014; Autmizguine et al., 2018). The signs of the disease are usually subtle, and despite the existence of effective antifungal treatments, the consequences for preterm infants are severe (Aliaga et al., 2014; Autmizguine et al., 2018). After *Candida* infection, almost 70% of infants born with birth weights under 1000 g lost their lives or developed severe neurodevelopmental disorders (Benjamin, 2006). Therefore, reducing the risk of invasive fungal infection in preterm infants is crucial.

The heightened vulnerability of hospitalized preterm infants to infection means that they often undergo long-term exposure to antibiotics. Increasing evidence shows that some specific antibiotics are associated with significantly increased risks of neonatal IFD (Benjamin, 2006; Lee et al., 2013; Fu et al., 2018; Warris et al., 2020; Kilpatrick et al., 2021). For example, a study of 3702 infants demonstrated that in extremely low birth-weight infants, long-term use of broad-spectrum antibiotics for at least 3 days after birth was associated with invasive candidiasis. This effect was especially strong for the use of third-generation cephalosporins, with a correlation coefficient of 0.67 (*P* = 0.017) (Cotten et al., 2006). In another study, 691 neonates who had been exposed to third-generation cephalosporins were found to be infected with *Candida*, which indicated a strong correlation between cephalosporins use and the incidence of invasive candidiasis (Benjamin, 2006). Thus, these studies suggest that either genetic or pharmacological interference with the host microbiota or the microbial environment may destroy the delicate balance and lead to the invasive growth of *Candida albicans* (*C. albicans*), causing candidiasis (Gutierrez et al., 2020; Costantini et al., 2022; Qin et al., 2022). However, while antibiotics are generally thought to increase the risk of fungal infection, the specific mechanisms leading to this increased risk are not entirely clear.

The microbiota during early life is important for infant health (Gasparrini et al., 2019; Rao et al., 2021). Importantly, the presence and activity of *C. albicans* have been found to be related to imbalances of the intestinal microbiome that can lead to health disturbances (Fox et al., 2014; Fan et al., 2015; Bergeron et al., 2017; Nogueira et al., 2019). Most studies of bacterial-fungal interactions have focused on interactions between pathogenic *C. albicans* and various bacteria (Nogueira et al., 2019), such as *Pseudomonas aeruginosa* (Bergeron et al., 2017), *Bacteroides fragilis* (Valentine et al., 2019), *Clostridium* species (Fan et al., 2015) and *Enterococcus faecalis* (Brown et al., 2019). IFD and the resulting microbial disruptions caused by various *Candida* species have been found to be associated with several factors. For instance, IFD is considered to be more likely to occur in patients with low immune function (Baptista et al., 2016; Brown et al., 2019; Ferreras-Antolin et al., 2019). In addition, either gestational age, mode of antibiotic treatment, host epithelial barrier function, immune ontogeny or diet may affect the composition of the microbiota (La Rosa et al., 2014; Bäckhed et al., 2015; Bokulich et al., 2016; DiBartolomeo and Claud, 2016; Gibson et al., 2016; Gregory et al., 2016; Shao et al., 2019; Ronan et al., 2021). However, due to these complexities, the impact of any single factor on microbiota development remains unclear, and understanding how antibiotics affect the microbiota of preterm infants and whether they promote fungal infection is a major challenge.

In order to clarify mechanisms by which antibiotics affect the microbiota and risk of IFD, we compared the clinical characteristics of fungus-infected and non-fungus-infected newborns after antibiotic treatment. The feces of these volunteers were studied through gene sequencing and analyses of 16S rRNA, and the compositions of their intestinal microbiomes were compared. Then, we constructed a rat fungal infection model and compared the responses of the microbiomes of rats with and without *C. albicans* infection to treatment with antibiotics. Finally, by using intestinal tissue immunofluorescence assays, the distribution and expression of IgA and IL10, the key molecules of immune regulation in the cecum, were detected. In summary, through this study, we aimed at elucidating the possible mechanisms of antibiotic-induced fungal infection in preterm infants.

## Materials and methods

### Participants

This retrospective study enrolled infants who were patients of the Guangzhou Women and Children Medical Care Center from January 2019 to December 2019. Three infants with clinically diagnosed fungal infection who had been admitted to the NICU constituted the fungal group, five infants who were treated with antibiotics but were not diagnosed with fungal infection constituted the non-fungal group, and four infants who were not treated with antibiotics and were not diagnosed with fungal infection constituted the control group. The inclusion criterion for patients with fungal infection was positive symptoms of fungal infection and an increased serum (1,3)-β-d-glucan level as confirmed with multiple blood tests. The inclusion criteria for the non-fungal group were as follows: admission to the NICU during the same time period, antibiotic use after birth due to other diseases, and without symptoms of fungal infection. The criteria for the control group were as follows: admission to the NICU during the same time period, no antibiotic use during hospitalization, and no evidence of fungal infection. All the studies followed the guidelines for the ethical treatment of human specimens and were approved by the Ethics Committee of Guangzhou Women and Children’s Medical Center. According to the Declaration of Helsinki, written informed consent was obtained from the parents of all enrolled neonates.

We recorded the general clinical data of all infants in the study, including sex, gestational age, birth weight, mode of delivery, premature rupture of membranes, 5 min Apgar score, and other diseases. The Apgar score comprises 5 components: (1) color, (2) heart rate, (3) reflexes, (4) muscle tone, and (5) respiration (Watterberg et al., 2015). The Apgar score was used to evaluate neonatal asphyxia. We also recorded additional clinical data of fungal group, including the types and days of antibiotic treatments, serum 1,3-β-D-glucan levels during hospitalization, the types and days of antifungal drugs, and the amount and duration of daily feeding.

### Collection of feces

Samples were taken from the fresh stool by using a sampling spoon, and the stool samples were quickly placed in a sterilized microcentrifuge tube. Then, the labeled tubes were transferred to a -80°C freezer for storage. After the diagnosis of IFD, the fecal samples of neonates in the fungal group were collected before antifungal treatment. The fecal samples of neonates in the non-fungal group were collected at the end of antibiotic treatment. The fecal samples of the control group were collected under normal feeding.

### Establishment of animal models

Animals in this study were approved by Guangzhou Medical University’s Institutional Animal Care and Use Committee and conducted in accordance with institutional guidelines. SPF-grade 0 to 2 day-old Sprague-Dawley (SD) rats, male or female, weighing 6 to 10 g, were provided by the Experimental Animal Center of Southern Medical University.

Increased risk of IFD in neonates has been associated with broad-spectrum antibiotic exposure, in particular with exposure to carbapenems (Fu et al., 2016; Esaiassen et al., 2017; Jiang et al., 2020). Therefore, we used meropenem in the establishment of a rat model of IFD. As in our ward, the infants who underwent co-administration of meropenem and vancomycin were more susceptible to IFD, we also included vancomycin in this treatment. In the fungal infection group (n = 9), the body weight (g) of rats were measured daily, and meropenem (2.4 mg/g) and vancomycin (0.9 mg/g) were injected intramuscularly once per day for 14 days. An intraperitoneal injection of 0.3 mL of *C. albicans* inoculum (10^8^ CFU/mL) was given on the 15th day. Saline solution was utilized in the vehicle group (n = 7) for 14 days, and then *C. albicans* inoculum was administered on the 15th day. Rats in the control group (n = 4) received regular feedings.

### Animal sampling

Fresh fecal pellets were taken from each rat on the 10th day after injection of *C. albicans*. Fresh fecal samples were obtained by placing a rat in an empty cage for a period of time during which its feces were excreted. The collected fecal pellets were put into a sterilized microcentrifuge tube and quickly stored at -80°C. All rats were sacrificed by cervical dislocation on the 10th day, and the severity of fungal infection was observed and intestinal samples were collected.

### Immunofluorescence staining

Sections of frozen ileum tissue (7 mm) were prepared and fixed with 4% PFA. Goat serum was added for 1 hour at room temperature, then the primary antibodies were applied overnight in a wet chamber at 4°C. Following the washing with PBS, the sections were incubated for 1 hour at room temperature with secondary antibodies and mounted them with VECTASHIELD Antifade Mounting Medium with DAPI to stain the nucleus. Using a Leica TCS SP8 Inverted Fluorescence Microscope (Leica Microsystems), immunofluorescent images were acquired. ImageJ software (National Institutes of Health, Bethesda, MD) was used to calculate the average fluorescence intensity of IgA and IL-10, and the number of CD11c clusters per square millimeter.

The primary antibodies and their concentrations were as follows: anti-IL-10 (bs-0698r, Bioss, China) (1:100), anti-IgA (MARA-1, Origene, USA) (1:100), and anti-CD11c (orb621157, Biorbyt, Cambridge, UK) (1:50). The secondary antibodies and their concentrations were as follows: goat anti-mouse IgG H&L-Cy3 (GB21301, Servicebio, China) (1:500), goat anti-rabbit IgG H&L-Cy3 (GB21303, Servicebio, China) (1:500), and goat anti-mouse IgG H&L-FITC (GB22301, Servicebio, China) (1:500).

### 16S rRNA analyses

According to the manufacturer’s instructions, DNA was extracted from microbial community using MagPure Stool DNA KF kit B (Magen, China). Degenerate PCR primers were used for amplification of variable region V4 of the bacterial 16S rRNA gene, primers 515F (5’-GTGCCAGCMGCCGCGGTAA-3’) and 806R (5’-GGACTACHVGGGTWTCTAAT-3’). PCR cycling conditions were 95°C for 3 min, followed by 30 cycles of 95°C for 45 s, 56°C for 45 s, and 72°C for 45 s, with a final extension of 10 minutes at 72°C. We purified the PCR products using Agencourt AMPure XP beads, followed by elution in elution buffer. The Agilent Technologies 2100 bioanalyzer was used to qualify libraries. In the sequencing process, the validated libraries were analyzed using an Illumina HiSeq 2500 platform (BGI, Shenzhen, China) following the standard Illumina pipelines, and two 250 bp paired-end reads were obtained.

Then, according to the 97% similarity criterion, we use *de novo* OTU screening with USEARCH (v7.0.1090) software platform to group reads into discrete operational taxonomic unit (OTU) clusters (Caporaso et al., 2010). These clusters were classified taxonomically using the Ribosomal Database Project (RDP; http://rdp.cme.msu.edu/) for bacteria. Based on annotations of OTUs, we generated phylogenetic relative abundance profiles at multiple taxa levels (phylum, class, order, family and genus). The Shannon diversity index, Simpson diversity index, and Chao1 diversity were used to estimate Alpha diversity. A weighted UniFrac distance was calculated using the QIIME (v1.80) pipeline to determine beta diversity (Edgar, 2010). R software (v3.1.1) was used to draw rank-abundance (Alroy, 2015) and Venn diagram analysis (Chen and Boutros, 2011). R software (v3.1.1, ade4 package) was used to draw PCA analysis. R software (v3.1.1, vegan package) was used to draw Nonmetric Multidimensional Scaling (NMDS) analysis. R software (v3.4.1) was used to draw a Spearman correlation heat map between dominant microbiome. PICRUSt2 software package (v2.2.0-b) and Kyoto Encyclopedia of Genes and Genomes (KEGG) database (http://geneontology.org/) were used to predict the functional content of microbial communities as KEGG ortholog profiles (Kanehisa et al., 2012). After obtaining the different gene functional pathways (Kruskal-Wallis tests), GraphPad Prism 8 (GraphPad Software Inc., USA) was used to represent data graphically. Linear discriminant analysis (LDA) effect size (LEfSe) was applied to find significant microbiome of different groups (Segata et al., 2011). Only taxa with LDA >3 at a P value <0.05 were considered significantly enriched.

### Statistical analysis

SPSS 25.0 software (SPSS, Inc., Chicago, IL, USA) and GraphPad Prism 8 (GraphPad Software Inc., USA) were used for statistical analyses. All data were first tested for normality and homogeneity of variance, and were statistically analyzed by analysis of variance. Composition and diversity of the gut microbiota were reported as mean ± SEM. The nonparametric Mann-Whitney U and Kruskal-Wallis tests were used for comparison of these factors. A permutational multivariate ANOVA PERMANOVA was used to test PCoA comparisons. NMDS comparisons were performed using ANOSIM. P < 0.05 was considered statistically significant.

## Results

### Clinical characteristics of premature infants with invasive fungal infections

This study involved a retrospective analysis of patients at our hospital. We collected the clinical information from three groups of infants. One group of infants (fungal group, n = 3) was diagnosed with fungal infections after being treated with broad-spectrum antibiotics for bacterial sepsis. A second group of infants (non-fungal group, n = 5) did not have any symptoms of fungal infection after broad-spectrum antibiotics therapy for bacterial sepsis. We identified a third group of infants (control group, n = 4) who did not have symptoms of fungal or bacterial infections, and were not treated with antibiotics. Fungal infections were diagnosed by combining clinical characteristics and blood levels of 1,3-β-D-glucan according to standard guidelines (Calley and Warris, 2017; King et al., 2017; Clancy and Nguyen, 2018; Warris et al., 2019; Weimer et al., 2021). We found that the average gestational age of infants with fungal infection was less than 30 weeks (29.5 ± 0.7 weeks) and the birth weight was less than 1500 g (1120 ± 226 g) (**Table 1**). There was no significant difference in the premature rupture of membranes, mode of delivery or the age of achieving full enteral feeding (*P* > 0.05) (**Table 1**).

**Table 1:** Comparison of clinical data among healthy volunteers, infants with fungal infection and infants without fungal infection (n=12).

Clinical information	Control group n=4	Fungal group n=3	Non-fungal group n=5	P value
Gestational age (weeks)	38.80 ± 1.3	29.5 ± 0.8^#^	35.6 ± 4.7	0.013
Birth weight (g)	3105 ± 346.1	1130.0 ± 226.1^#^	2072.0 ± 1016.8	0.017
Male infants (n)	2	3	1	0.134
Caesarean section (n)	0	1	1	0.697
Premature rupture of membrane (n)	1	1	0	0.470
Apgar score ≤7 at 5 minutes (n)	0	1	0	0.250
Age of complete feeding^&^ (days)	*	50.67 ± 18.93	22.0 ± 16.6	0.064^+^

*The patients had achieved the full enteral feeding when they were admitted to the NICU. ^&^ A newborn feeding amount of 150ml/kg per day means complete feeding. The p value was analyzed by One-way ANOVA or fisher exact test. #P<0.05 vs Control. +P value was analyzed by two-tailed Student t test. The data are presented as the mean ± SD.

We found that patients diagnosed with a fungal infection tended to have received injections of broad-spectrum antibiotics for more than 2 weeks prior to the fungal infection. In two cases, the treatment lasted for more than 1 month (**Table 2**). The average exposure time to broad-spectrum antibiotics in the fungal group was longer than that in the non-fungal group. All of the cases in fungal group received carbapenem therapy. The group of infants with fungal infection had more severe complications than those in the non-fungal group and the control group (**Table 2**).

**Table 2:** Clinical and laboratory information among the three groups of individuals (n=12).

Groups	Fungal group (F1-3)			Non-Fungal group (N1-5)					Control group(C1-4)			
Infants	F1	F2	F3	N1	N2	N3	N4	N5	C1	C2	C3	C4
Types and duration of antibiotics before fungal infection (d)	Carbapenems (19)	Carbapenems (12) Penicillins (1) Glycopeptides (10) Cephalosporins (9)	Carbapenems (44) Penicillins (37)	Penicillins (10)	Carbapenems (33) Penicillins (6) Glycopeptides (14) Cephalosporins (46)	Penicillins (17) Cephalosporins (30)	Cephalosporins (6)	Carbapenems (17)	–	–	–	–
Age of fungal infection onset (d)	19	27	131	–	–	–	–	–	–	–	–	–
Level of 1,3-β-d-glucan before using antifungal drugs (pg/ml)	152.53	189.37	>600	–	–	–	–	–	–	–	–	–
Categories and duration of antifungal drugs (d)	Azoles (47)	Azoles (77)	Azoles (3)	–	–	–	–	–	–	–	–	–
Complication	Encephalopathy, Pneumonia, Atelectasis, ROP*, NEC*	Pneumonia, Laryngomalacia, Gastroesophageal reflux, Encephalopathy, BPD*	RDS*, Sepsis, Pneumonia, BPD*, PDA*, IVH*, Hyperbilirubinemia	Intraventricular hemorrhage, Ischemic stroke	BPD*, RDS*, Intraventricular hemorrhage, HIE*, Sepsis	Congenital retinitis pigmentosa, Albinism	Pneumonia, Cleft palate, Pierre Robin sequence	Intraventricular hemorrhage, Subdural hematoma, Skull fractures	–	–	–	–

*NEC, Neonatal necrotizing enterocolitis; RDS, Respiratory distress syndrome; ROP, Retinopathy of Prematurity; HIE, Hypoxic-ischemic encephalopathy; BPD, Bronchopulmonary Dysplasia; PDA, Patent ductus arteriosus; IVH, Intraventricular hemorrhage. F1-3 stand for patients in fungal group, N1-5 stand for patients in non-fungal group, C1-4 stand for patients in control group.

**Figure 1:**
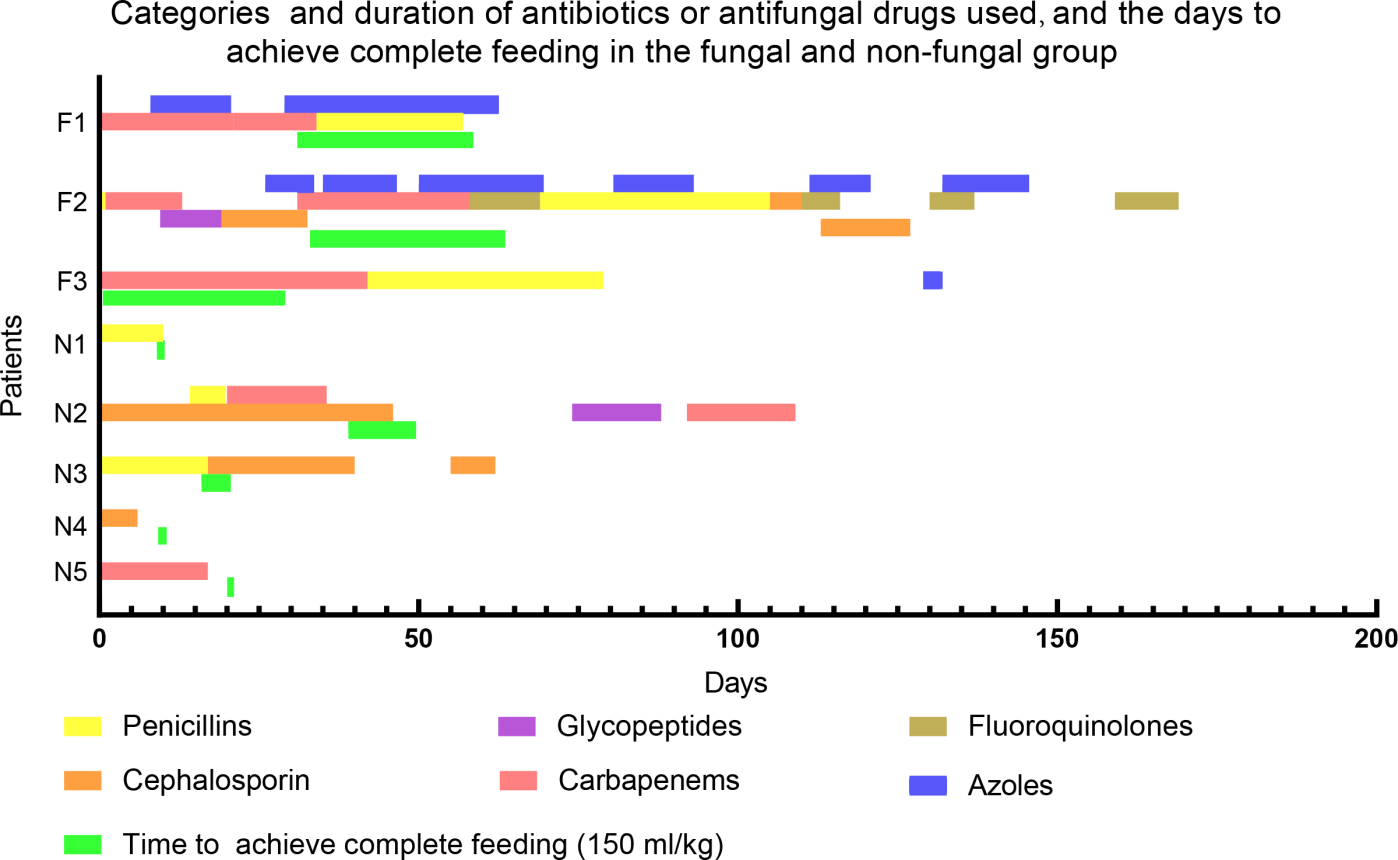
Categories and duration of antibiotics or antifungal drugs used, and the time to achieve complete feeding in the fungal and non-fungal group. F1-3 stand for patients in fungal group, N1-5 stand for patients in non-fungal group. A newborn feeding amount of 150ml/kg per day means complete feeding.

Previous studies demonstrated that low birth weight, use of broad-spectrum antibiotics (e.g. third-generation cephalosporins or carbapenems) for greater than 7 days, mechanical ventilation, central vascular catheter, and delayed full feeding were risk factors of invasive fungal infections (Feja et al., 2005; Hsieh et al., 2012; Lee et al., 2013; King et al., 2017). These findings are consistent with our results regarding the characteristics of the fungal group. However, we noticed that two infants in the non-fungal group who had been exposed to third-generation cephalosporins or carbapenems for more than one month had no invasive fungal infections. We further found that the time required for infants in the fungal group to achieve total enteral feeding was longer than that in the non-fungal group (50.67 ± 18.93 vs 22.0 ± 16.6 days, **Table 1**; **Figure 1**). Thus, there were significant differences in specific tested clinical parameters between fungal and non-fungal group.

### Characteristics of intestinal microbiomes in neonates with *C. albicans* infection after antibiotic use

It has been shown that the type of birth, gestational age, the type of feeding, and antibiotic treatment affect the microbial colonization of preterm infants (Parra-Llorca et al., 2018; Zwittink et al., 2018; Healy et al., 2022). Therefore, we collected the feces of these three groups of infants and analyzed the intestinal microbiomes in order to explore the relationship between the intestinal microbiome and invasive fungal infection. After performing quality control of the sequences, a total of 704,264 high-quality sequences were obtained from all patient samples.

**Figure 2:**
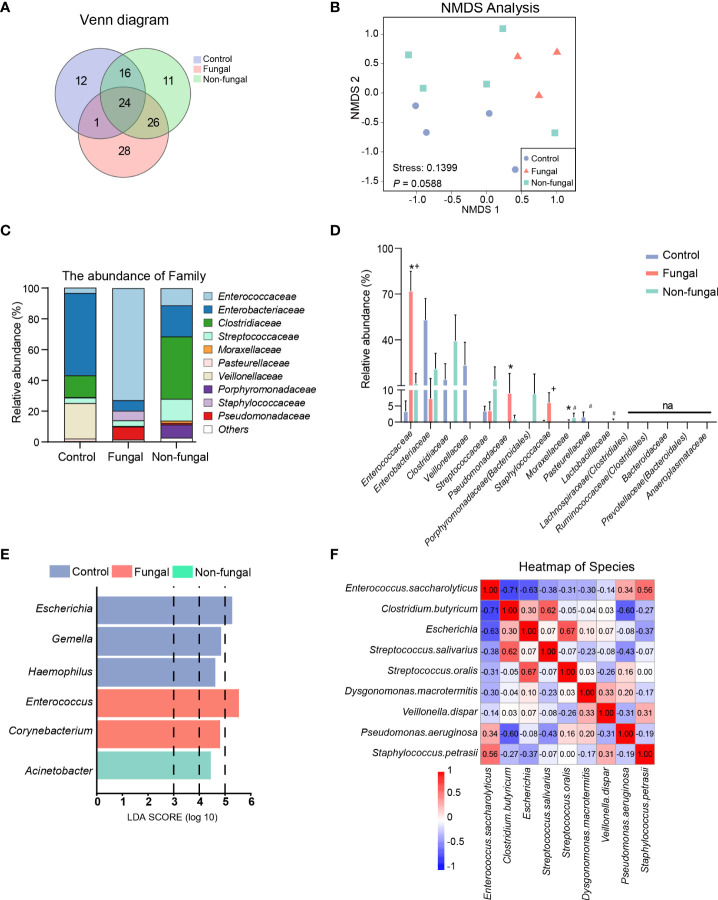
Evaluation of microbiome composition in newborns without antibiotics and without fungal infection (control group), newborns treated with antibiotics (non-fungal group) and newborns infected with fungi (fungal group). **(A)** A Venn diagram shows the common and unique OTUs of the three groups, where Core represents the common OTUs of the three groups. **(B)** Nonmetric Multidimensional Scaling (NMDS): groups were compared using ANOSIM. Relative proportions of sequences read at the family **(C)** levels assigned to different bacteria. The microbiome abundance was less than 0.5% in all samples, and the unannotated microbiomes were all merged into Others. Comparison of relative abundance of bacteria at family **(D)** levels. The results are mean ± SEM, and the P values of the differences between groups were tested by the Mann-Whitney U test. *P < 0.05 vs Control, #P < 0.05 vs Control, +P < 0.05 vs Fungal. **(E)** Differential enrichment of intestinal microbiota of rats in each group at the genus level according to linear discriminant analysis (LDA). Only taxa with LDA >3 at a P value <0.05 were considered significantly enriched. **(F)** Heat map: the number in the box is the Spearman coefficient, whose color deepens with the increase of the absolute value. Spearman values range from -1 (blue) to 1 (red).

By applying the USEARCH software platform, OTUs clusters were identified based on nonrepetitive sequences. 53, 77 and 79 OTUs were found from the control group, non-fungal group and fungal group, respectively. The analysis also showed that there were 24 identical OTUs in the three groups, and 28 OTUs were identified as being specific to the fungal group (**Figure 2A**).

We evaluated the composition of the microbiome communities in the intestines of these infants by calculating alpha diversity and beta diversity. Regarding alpha diversity, the increase of the Chao1 index represents the richness of the intestinal bacterial community, and a lower Simpson index or higher Shannon index suggests a higher diversity of intestinal bacterial communities. The fungal group showed more estimated richness according to the Chao1 index than did the control group, but the differences between these groups did not rise to the level of statistical significance (**sFigure1A**). The Shannon index of the fungal group was decreased (**sFigure1B**), while the Simpson index was increased (**sFigure1C**), suggesting that the diversity of the microbiome changed upon infection, but there was no significant difference between the groups.

Next, we estimated beta diversity of gut microbiota based on their relative abundances and shared OTUs. A Principal Component Analysis (PCA) illustrated the similarities and differences of the three groups of bacteria based on the total number of OTUs. As shown in **sFigure1D**, there were some notable separations among the microbial populations of the fungal group, the control group and the non-fungal group. The percentages attributed to variations in Principle Component (PC)1 and PC2 were 38.15% and 22.08%, respectively. NMDS (**Figure 2B**) provided another method to identify the similarities and differences among the three groups; in this analysis, the stress coefficient was found to have explanatory significance (stress < 0.2, *P* = 0.0588, ANOSIM).

According to the absolute abundances of OTUs and the species annotation information, the microbiota compositions of each group at the family and genus levels were analyzed statistically. At the family level (**Figures 2C, D**), the composition of the intestinal microbiome in neonates with fungal infections was clearly altered. Interestingly, the relative abundance of inflammation-associated microbiota, including *Enterococcaceae* (72.00 vs 3.30%, *P* = 0.034) and *Pseudomonadaceae* (9.04 vs 0%, *P* = 0.028) in infants with a fungal infection was significantly higher than that of control group. This difference was especially clear with the *Enterococcaceae* family. The abundance of *Enterobacteriaceae* was decreased in the fungal group and non-fungal group relative to the control group, and it was especially decreased in the fungal group. *Clostridiaceae* was almost absent from the fungal group, but it was abundant in the non-fungal group. *Veillonellaceae* and *Pasteurellaceae* were detected at low levels in the fungal and non-fungal group.

These major changes observed at the family level were similarly observed in downstream taxa. At the genus level (**sFigures 1E, F**), we confirmed that *Enterococcus* (72.01 vs 3.30%, *P* = 0.034) and *Pseudomonas* (9.04 vs 0%, *P* = 0.028) were enriched in fungal group compared to control group, while the number of species of the genus *Escherichia* (*Enterobacteriaceae*) (0 vs 36.77%, *P* =0.028) was lower. *Clostridium_sensu_stricto*, which are linked to the production of butyric acid, almost disappeared in the intestines of the three patients in the fungal group. Although the patient sample size was small, clear trends indicating a decrease of *Clostridiaceae* and increase of *Enterococcaceae* in the fungal infection group were noted.

A GraPhlAn plot was created to show the overall composition of the microbiota at the phylum-to-genus level of all samples. We observed that the abundance of *Enterococcus* was higher in the fungal group and the abundance of *Enterobacteriaceae* and *Clostridiaceae* were enriched in the control group and non-fungal group (**sFigure 1G**). The distribution of enriched bacteria at the genus level was identified using LEfSe analysis (**Figure 2E** and **Supplement 1**). Although the patient sample size was small, an enrichment of *Enterococcus* (LDA = 5.55, *P* = 0.024) in the fungal group attracted our attention. A heat map based on the Spearman coefficient showed the correlation between the microbiome at the species level; we found that there was a strong negative correlation between *Enterococcus* and *Clostridium butyricum* (*C. butyricum*), with a Spearman coefficient of -0.71 (**Figure 2F**).

**Figure 3:**
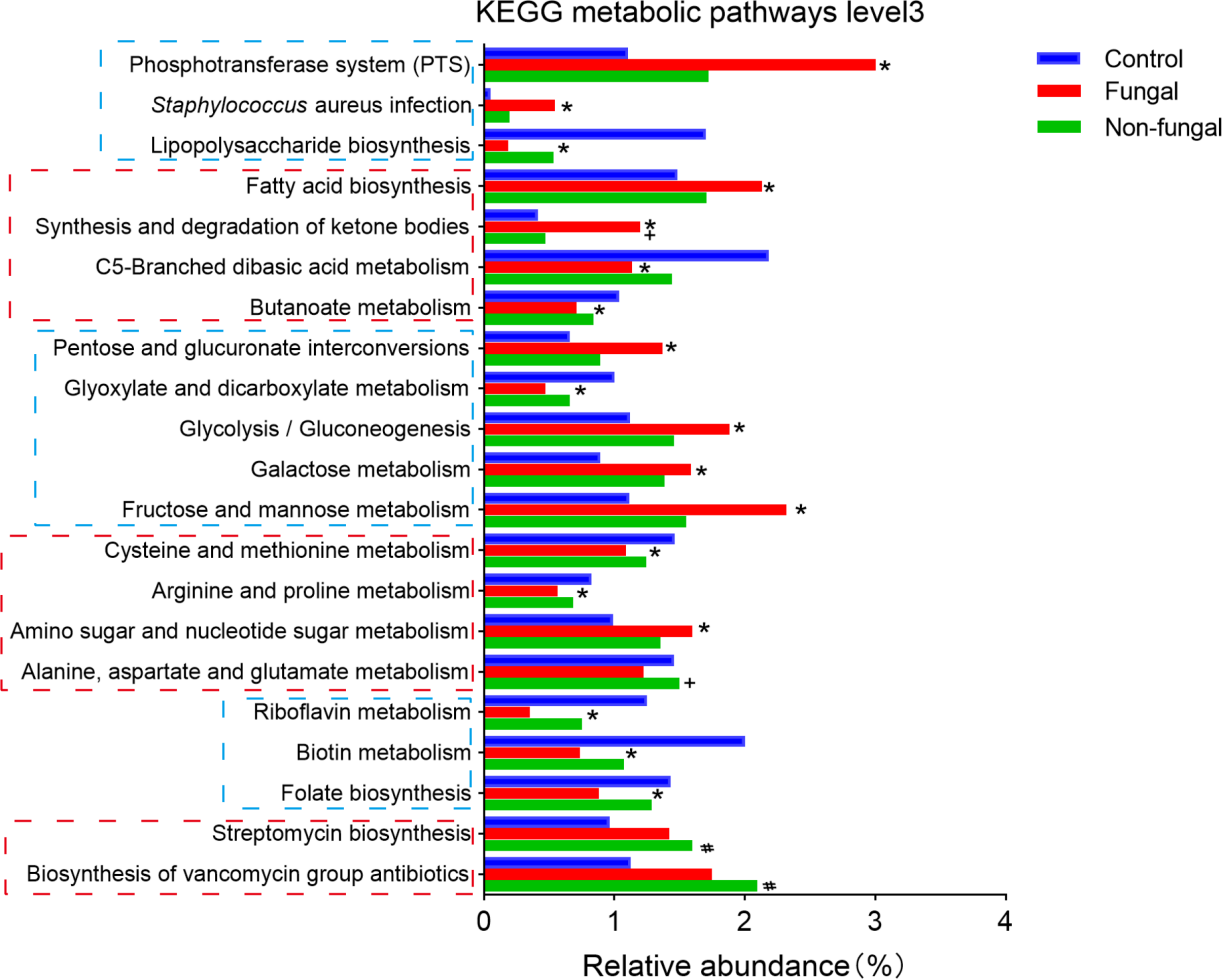
Differences of neonates in intestinal KEGG metabolic pathways level 3. *P < 0.05 vs Control, #P < 0.05 vs Control, +P < 0.05 vs Fungal. The P value was calculated with the Kruskal–Wallis test.

Then, we used KEGG analysis to predict metabolic pathways of the infant intestinal microbiome (**Figure 3**). At the third hierarchical level of KEGG pathways (KEGG level 3), the metabolism of vancomycin (1.75 and 2.09 vs 1.13) and streptomycin (1.42 and 1.60 vs 0.96) were predicted to increase significantly in the fungal group and the non-fungal group compared to control group; these changes were likely related to the use of antibiotics in these neonates. In addition, bacterial infection (0.54 and 0.20 vs 0.05) and bacterial phosphotransferase system (3.0 and 1.72 vs 1.11) pathways were significantly enriched in the fungal and non-fungal groups. Glucose metabolism, amino acid metabolism and vitamin metabolism in the fungal and non-fungal group were disordered. Interestingly, butanoate metabolism (0.71 vs 1.04) decreased significantly in the fungal group compared to control group, while the demand for fatty acid (2.13 vs 1.49) and ketone synthesis (1.20 vs 0.41) increased.

### Association of *C. albicans* infection with gut microbial changes in a rat model

Based on the strong negative correlation between *Enterococcus* and *C. butyricum* in intestinal microbiomes of infants infected with fungi, we hypothesized that these microbes play important roles in the process of neonatal fungal infection. Therefore, we constructed a rat fungal infection model to test our hypothesis. In a previous work, we showed that compared with untreated neonatal rats, the immunity of neonatal rats to *C. albicans* infection was significantly impaired after antibiotic treatment. This damage was manifested by increased levels of fungal glucan in peripheral blood, intestinal congestion, ischemia, multiple caseous fungal infections in the abdominal cavity and reduction of intestinal villi (Wang et al., 2020). These symptoms were similar to those of IFD.

**Figure 4:**
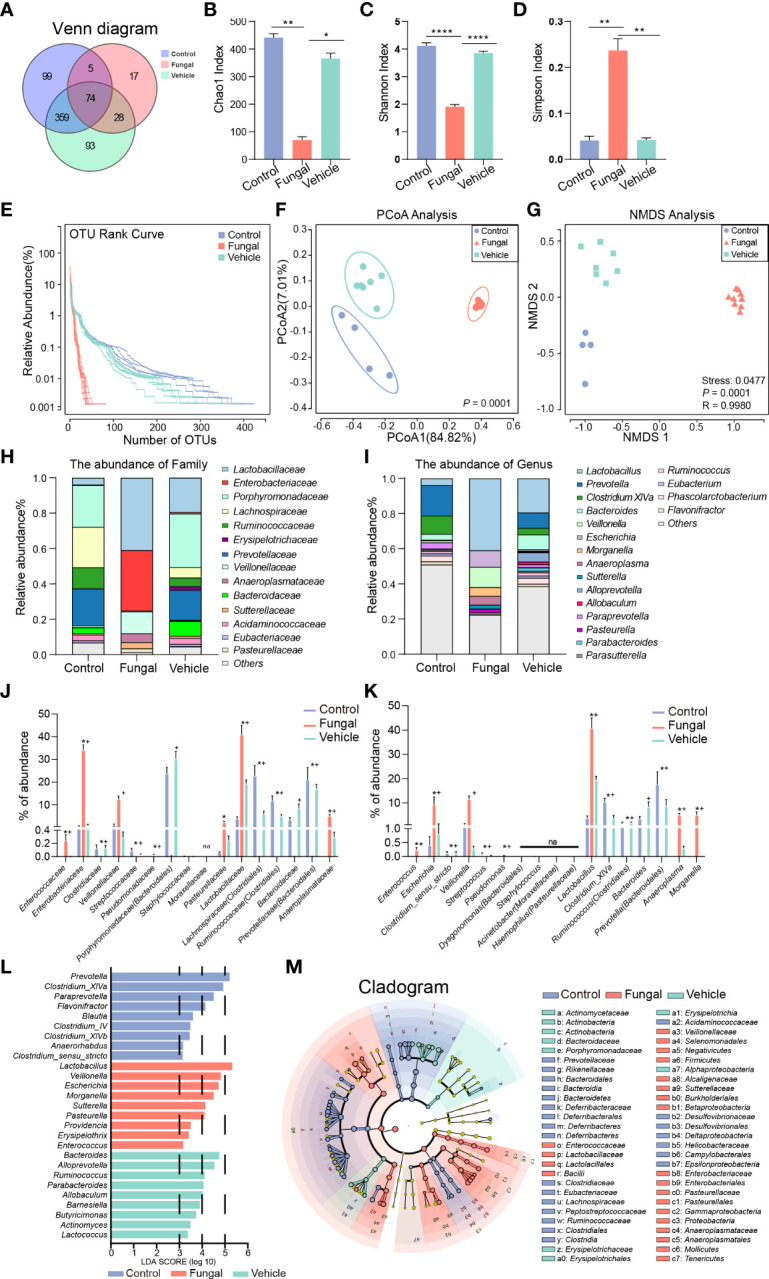
Comparison of intestinal microbial abundance and community composition among rats without any treatment (Control group), rats infected with fungi after antibiotic treatment (Fungal group) and rats inoculated with fungi only (Vehicle group). **(A)** A Venn diagram analysis shows the common and unique OTUs among the three groups. Three indexes of alpha diversity are displayed: **(B)** the Chao1 index, **(C)** the Shannon index, and **(D)** the Simpson index. The data are presented as the mean ± SEM. The P value was calculated with a Kruskal–Wallis test. *P < 0.05, **P < 0.01, ****P < 0.0001. **(E)** The RNK curve: the abscissa is sorted by sample OTUs, and the ordinate is OTU abundance. **(F)** Principal co-ordinate analysis (PCoA). PERMANOVA was used for comparison between groups. **(G)** Nonmetric Multidimensional Scaling (NMDS); groups were compared using Anosim. The Stress represents the difference between the distance of a point in two-dimensional space and that in multi-dimensional space. Stress < 0.05 is a perfect representation. Relative proportions of sequence read at the family **(H)** and genus **(I)** levels assigned to different bacteria. The microbiome abundance was less than 0.5% in all samples, and the unannotated microbiomes were all merged into Others. Comparison of relative abundance of bacteria at the family **(J)** and genus **(K)** levels. The results are presented as mean ± SEM, and the P value was calculated with a Kruskal–Wallis test. *P < 0.05 vs Control, #P < 0.05 vs Control, +P < 0.05 vs Fungal. **(L)** Differential enrichment of intestinal microbiota of rats in each group at the genus level according to linear discriminant analysis (LDA). Only taxa with LDA >3 at a P value <0.05 were considered significantly enriched. **(M)** Cladogram shows the community composition of the gut microbiota in rats based on LEfSe analysis.

Therefore, we further analyzed and evaluated the microbiome in the rat model of fungal infection. This analysis led to the identification of 537 OTUs in control rats (control group), 124 OTUs in rats infected with fungi after antibiotic treatment (fungal group) and 554 OTUs in rats infected with fungi without antibiotic treatment (vehicle group). We also identified 17 OTUs that were specific to the fungal group, indicating that the abundance and composition of microbial communities in the fungal group changed dramatically with antibiotic treatment (**Figure 4A**).

A detailed examination of the microbiota by investigation of alpha diversity and beta diversity showed important differences between groups. Regarding alpha diversity, the Chao1 index decreased sharply in the fungal group (**Figure 4B**), suggesting that the microbiome abundance decreased in this group. A decreased Shannon index (**Figure 4C**) and an increased Simpson index (**Figure 4D**) indicated that *C. albicans* infection significantly reduced the diversity of the intestinal microbiome in rats after antibiotic pretreatment. Upon rank-abundance curve analysis, which can reflect the richness and uniformity of sample microbiome, there was no significant difference found in the length and steepness of the curves between the control group and the vehicle group, while the curve of the fungal group was steep and the length was shortened. These rank-abundance curves reflected the fact that a few dominant phylotypes comprise the major proportion of microbial communities in the rats of the fungal group, whereas more communities were seen in rats of the control and vehicle groups (**Figure 4E**). A principle component analysis (PCoA) of weighted UniFrac distance identified a clear separation of the microbiomes of the three groups, and each group was highly aggregated (**Figure 4F**). The percentages attributed to variations in PCoA1 and PCoA2 were 84.82% and 27.01%, respectively. NMDS (**Figure 4G**) analysis also confirmed the noted differences among the three groups (stress = 0.0477, *P* < 0.001, ANOSIM).

Next, we analyzed differences in the rat gut microbial communities at the levels of the family and genus that were caused by *C. albicans* infection after antibiotic pretreatment. When comparing the abundances, we ranked the strains on the abscissa according to their abundances within the human intestinal microbiome (**Figure 2D**). Although the abundances and compositions of rat microbiome were not completely consistent with the human microbiome, this comparison can still emphasize similar trends of several bacteria.

At the family level (**Figures 4H, J**), *Clostridiaceae* (0.00 vs 0.12%, *P* =0.019), *Ruminococcaceae* (*Clostridiales*) (0.001 vs 11.78%, *P* < 0.001) and *Lachnospiraceae* (*Clostridiales*) (0.002 vs 22.74%, *P* < 0.001) nearly disappeared in the fungal group compared to the control group. This finding was consistent with the change of *Clostridiaceae* in neonates infected with fungi (**Figure 2D**). Interestingly, lower levels of *Bacteroidaceae* (0.003 vs 3.42%, *P* = 0.084), *Prevotellaceae* (*Bacteroidales*) (0.003 vs 21.03%, *P* = 0.006) and *Porphyromonadaceae* (*Bacteroidales*) (0.009 vs 23.61%, *P* = 0.088) and higher levels of *Enterococcaceae* (0.24 vs 0%, *P* = 0.005), *Enterobacteriaceae* (34.14 vs 0.40%, *P* = 0.028), *Veillonellaceae* (12.43 vs 1.13%, *P* = 0.088), *Lactobacillaceae* (40.80 vs 3.83%, *P* < 0.001), *Pasteurellaceae* (2.18 vs 0.06%, *P* = 0.014) and *Anaeroplasmataceae* (5.06 vs 0%, *P* < 0.001) were observed in the fungal group as compared to the control group.

We further investigated changes of the microbiomes at the genus level. At the genus level (**Figures 4I, K**), *Clostridium_XlVa* (0 vs 10.28%, *P* < 0.001), *Clostridium_sensu_stricto* (0.001 vs 0.12%, *P* = 0.019), *Ruminococcus (Clostridiales*) (0 vs 1.42%, *P* = 0.027) and *Prevotella* (*Bacteroidales*) (0.002 vs 17.52%, *P* = 0.003) nearly disappeared in the fungal group compared to the control group. We noted that *Lactobacillus* was significantly enriched in the fungal group and the vehicle group. *Lactobacillus* was the dominant resident bacteria in the control group, and the increase of its relative abundance seemed to be caused by decreases of other bacteria. In addition, higher levels of *Enterococcus* (0.24 vs 0%, *P* = 0.008), *Escherichia* (9.24 vs 0.40%, *P* = 0.044), *Veillonella* (11.53 vs 1.13%, *P* = 0.088), *Anaeroplasma* (5.06 vs 0%, *P* < 0.001) and *Morganella* (5.06 vs 0.001%, *P* = 0.028) were observed in the fungal group as compared to the control group.

The distribution of enriched bacteria was identified at the genus level using LEfSe analysis. The LDA scores of 9 differentially abundant taxa in the fungal group were higher than 3 (**Figures 4L, M**; **Supplement 1**). These taxa were *Lactobacillus*, *Veillonella*, *Escherichia*, *Morganella* (*Enterobacteriaceae*), *Sutterella*, *Pasteurella*, *Providencia* (*Enterobacteriaceae*), *Erysipelothrix* and *Enterococcus*. Compared with rats subjected to fungal infection, *Prevotella*, *Clostridium_XlVa*, *Paraprevotella*, *Flavonifractor* (*Ruminococcaceae*), *Blautia* (*Clostridiales*), *Clostridium_IV*, *Clostridium_XlVb*, *Anaerorhabdus* (*Bacteroidaceae*) and *Clostridium_sensu_stricto* had higher LDA scores in the control group. Most of these microbes are well known to produce short-chain fatty acids (SCFAs), such as acetic acid and butyric acid. *Enterococcus* (LDA = 3.20, *P* < 0.001) in the fungal group, *Clostridium_XlVa* (LDA = 4.93, *P* < 0.001) in the control group and *Bacteroides* (LDA = 4.78, *P* < 0.001) and *Ruminococcus* (LDA = 4.14, *P* < 0.001) in the vehicle group were other findings that attracted our attention. Taken together, we have found that the intestinal microbiomes of infants with clinical fungal infections and rats in a model of fungal infection were enriched in proinflammatory *Enterococcus* and decreased in SCFA-producing *Clostridium* species.

**Figure 5:**
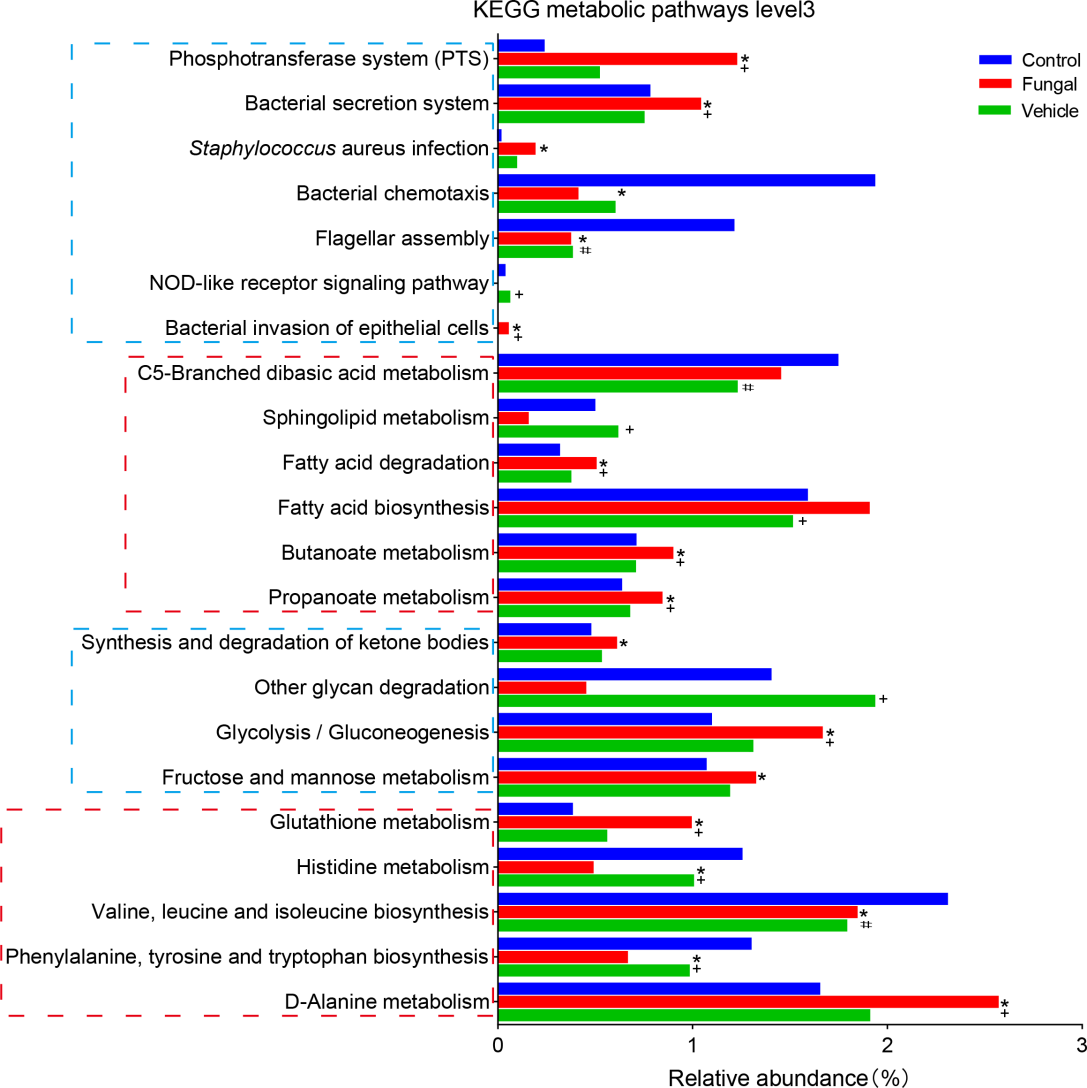
Differences in intestinal KEGG metabolic pathways level 3 among control rats, rats infected with fungi after antibiotic treatment and rats inoculated with fungi only. *P < 0.05 vs Control, #P < 0.05 vs Control, +P < 0.05 vs Fungal. The P value was calculated with a Kruskal–Wallis 4test.

Finally, we used PICRUST2 to predict the KEGG metabolic pathway level 3 of the intestinal microbiomes in the three groups of rats (**Figure 5**). The functions of amino acid metabolism and glucose metabolism in the fungal group were disordered. Compared with control group, *C. albicans* infection significantly increased the bacterial phosphotransferase system (1.23 vs 0.24), weakened bacterial chemotaxis (0.41 vs 1.94), and increased bacterial secretion (1.04 vs 0.78), invasion (0.06 vs 0.002) and infection (0.19 vs 0.02). Interestingly, pathways involving fatty acid synthesis (1.91 vs 1.59) and degradation (0.51 vs 0.32) increased in the fungal group compared to control group; these pathways included the metabolism of butyrate (0.91 vs 0.71), propionate (0.85 vs 0.64) and ketone bodies (0.61 vs 0.48). In addition, the normal activation of the NOD-like receptor signaling pathway (0.004 vs 0.04) in the intestinal microbiome of rats infected with *C. albicans* seemed to be inhibited.

### Association of *Clostridium* species with loss of intestinal immunosuppression

As mentioned above, the intestinal microbiomes of rats infected with fungi showed significant changes after antibiotic treatment. Increases of *Enterococccus* and other opportunistic pathogens can induce inflammatory intestinal injury (Fiore et al., 2019). Of more concern is the disappearance of the genera *Clostridium* and the decrease of *Ruminococcus*, *Lachnospiraceae* and *Bacteroidacea*. Bacteria from these genera are dominant commensal bacteria that are known to be producers of SCFAs, which tend to control inflammation (Fan et al., 2015; Stoeva et al., 2021). In particular, the butyrate molecule can be shown to be effective in maintaining T regulatory (Treg) cell differentiation (Furusawa et al., 2013).

**Figure 6:**
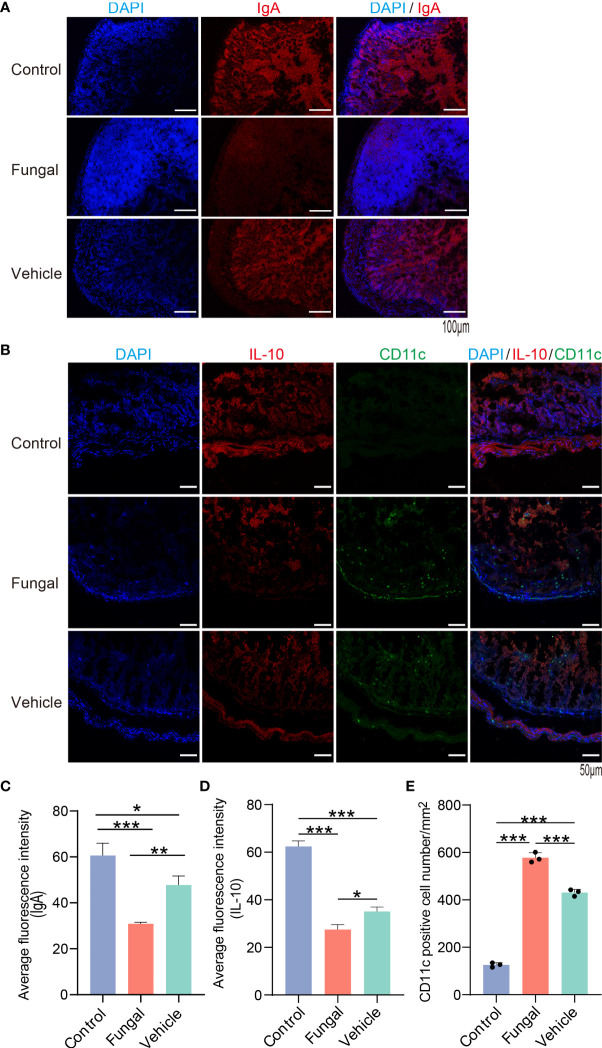
The expression of IL10 and IgA in intestinal tissues of rats with fungal infection decreased after antibiotic treatment. **(A)** IgA (red) and DAPI for nuclei (blue) and **(B)** IL10 (red), CD11c (green) and DAPI for nuclei (blue) in intestinal tissues of rats of the three group. **(C, D)**: The average fluorescence intensity of IgA and IL10. **(E)** Number of CD11c^+^ cells/mm^2^. *P < 0.05, **P < 0.01, ***P < 0.001, by one-way ANOVA with Tukey’s multiple comparisons test. Data are presented as the mean ± SD of at least 3 independent experiments.

SCFAs promote intestinal IgA immune function and induce intestinal IL-10 expression to play an anti-inflammatory role (Kanai et al., 2015; Wu et al., 2017; Ariyoshi et al., 2020). Pro-inflammatory CD11c^+^ macrophages produce cytokines that promote intestinal inflammation and mucosal injury, while CD11c^−^ macrophage-like cells produce IL-10 (Arnold et al., 2016; Girard-Madoux et al., 2016; Bernardo et al., 2018). Compared with the control group and vehicle group, the expression of IgA and IL10 in the intestinal tissue of rats infected with *C. albicans* pretreated with antibiotics was significantly decreased (**Figures 6A**–**D**). In addition, CD11c expression in proinflammatory macrophages increased significantly (**Figures 6B, E**). The microbiota metabolite butyrate promotes intestinal IgA immune function (Isobe et al., 2020). Butyrate-producing *C. butyricum* induces the increase of IL10 expression in the intestine to play an anti-inflammatory role (Kanai et al., 2015; Ariyoshi et al., 2020). Therefore, the susceptibility to fungal infections after antibiotic use may be due to dysregulation of mucosal immune regulatory molecules such as IL10 and IgA. SCFA-producing *Clostridium* species may also play a key role in this process.

## Discussion

**Figure 7:**
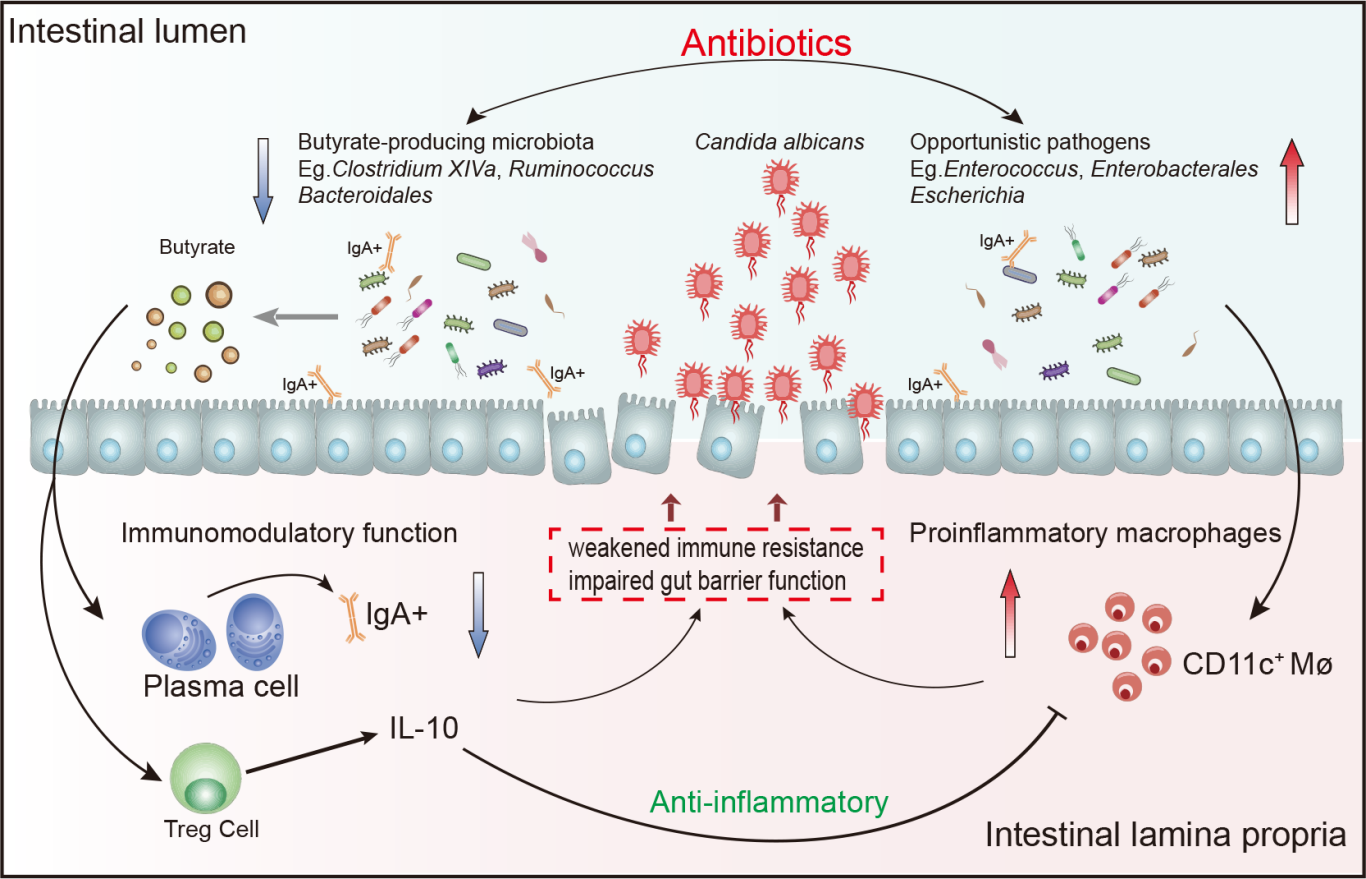
Schematic drawing showing relationship between the intestinal microbiome and immune imbalance.

Gut microbes play a crucial role in host health and disease of the host all through life, but its influence on diseases at the early stage of life is unclear. Neonatal IFD due to *Candida* is an important cause of morbidity and mortality in critical neonates, especially in premature infants (Benjamin, 2006; Kilpatrick et al., 2021). Using broad-spectrum antibiotics is a risk factor for neonatal *Candida* infection, because it can eliminate healthy bacterial microbiome and lead to fungal overgrowth (Cotten et al., 2006; Tripathi et al., 2012; Kilpatrick et al., 2021). Previous studies revealed that long-term exposure to antibiotics leads to a disordering of the intestinal microbiome that is mainly manifested as decreased probiotics and increased antibiotic-resistant pathogens (Tripathi et al., 2012; Gasparrini et al., 2019; Ramirez and Cantey, 2019), but the change in intestinal microbiome of neonates with IFD is unknown. In our study, clear trends indicating a decrease of *Clostridium* species in neonates with IFD was noted. We also confirmed that SCFA-producing *Clostridium* species almost disappeared from the intestinal microbiome of a rat model of IFD, while *Enterococcus* increased significantly. The expression of IgA and IL10 in the intestinal tissue of fungus-infected rats after antibiotic treatment decreased significantly, suggesting that antibiotics may affect intestinal immune homeostasis by changing neonatal intestinal microecology. The improper use of broad-spectrum antibiotics can lead to the increase of opportunistic pathogens and the depletion of SCFA-producing *Clostridium* species and *Bacteroides*, which can affect immune homeostasis and induce IFD (**Figure 7**).

We found that the levels of *Clostridium* species and *Bacteroidales*, which produce SCFA (including acetic acid and butyric acid), decreased sharply in both IFD infants and rats. *Clostridium _ XlVa* and *Bacteroides* have been confirmed to activate HIF-1α and IL-37 to inhibit the colonization of *C. albicans* (Fan et al., 2015). Moreover, animal studies revealed that *Clostridium* species clusters IV, XIVa and XVIII are involved in SCFA production and have been demonstrated to promote the induction of colonic Tregs (Atarashi et al., 2011; 2013). The gut microbiome regulates the effects of local and systemic immunity through SCFAs, especially butyrate (Markowiak-Kopec and Slizewska, 2020). The gut epithelial cells use butyric acid as 70% of their energy to regulate immune cells and anti-inflammatory factors (such as IL-10, IgA and TGF-β) (Siddiqui and Cresci, 2021). In this study, we found a significant depletion of *C. butyricum* in the gut microbiota of IFD rats. According to the KEGG metabolic pathway, abnormalities in pathways related to the metabolism of SCFAs (including butyric acid, propionic acid and ketone bodies) were related to alterations in the abundance of these probiotics that produce SCFAs. We believe that the depletion of *Clostridium* species weakened the resistance of rats to fungal colonization.

*Enterococcus* naturally exists in the environment and is an important component of human and animal intestinal microbiomes (Wada et al., 2019). After the use of antibiotics, however, the number of *Enterococci* will likely continue to increase, which may be the key event to induce fungal infection after the use of antibiotics (Elvers et al., 2020). At the same time, drug-resistant *Enterococcus* is one of the severe challenges faced in the NICU, as this organism causes immune-mediated inflammatory injury to the intestinal mucosal (Arias and Murray, 2012; Fiore et al., 2019). For the first time, we found an increase in the abundance of *Enterococcus* in the intestinal tracts of infants infected with fungi after the use of antibiotics. Similarly, a significant increase in opportunistic pathogens, including *Enterococcus*, was clearly demonstrated in rats infected with fungi after antibiotic use. We also found a strong negative correlation between *Enterococcus* and *C. butyricum* in IFD rats. We consider that the increased *Enterococcus* may take part in the susceptibility to fungi after the use of antibiotics.

The gut and other mucosal tissues are rich in IgA (Pietrzak et al., 2020). As part of mucosal immunity, secretory IgA is involved in pathogen elimination and the regulation of the intestinal microbiome (Tsuji et al., 2008; Pabst and Slack, 2020; Rollenske et al., 2021). For example, *Bacteroides* can induce the production of IgA in the large intestine through the formation of germinal centers and an increase in the number of B cells that produce IgA (Yanagibashi et al., 2013). In this study, it was confirmed that *Clostridium* species and *Bacteroides* are decreased and *Enterococcus* are increased after antibiotic use, and these are closely related to the key proteins IgA and IL10 in the maintenance of intestinal homeostasis. We considered that the decrease in intestinal immune resistance mediated by SCFA promoted the colonization of invasive fungi. In the future, we plan to quantify changes to the butyric acid content and to further test its role in resisting fungal colonization.

The data in this study show that one of the most important preventive measures is to avoid the irregular use of broad-spectrum antibiotics in order to protect the appropriate intestinal microbiome. In addition, *Clostridium* probiotics and their metabolites have been shown to enhance the gastrointestinal mucosal barrier and help regulate Treg cell immune responses (Atarashi et al., 2013; Cao et al., 2019; Liu et al., 2019; Guo et al., 2020; Hagihara et al., 2020). Therefore, we propose that reintroducing symbiotic microbiota may reduce the risk of IFD. However, a limitation of this study is that the sample size was small, and further exploration of underlying mechanisms was not conducted. In the future, genetically engineered rats and aseptic rats will be used to further explore the molecular mechanisms to clarify the relationships between specific bacteria and intestinal immunity.

## Conclusion

In summary, the gut microbiota is highly affected by the use of antibiotics and is closely related to the pathogenesis of IFD. The inappropriate use of broad-spectrum antibiotics can lead to the increase of opportunistic pathogens and the depletion of *Clostridium* species and *Bacteroides* in preterm infants. The depletion of these SCFA-producing bacteria ultimately reduces the IgA- and IL-10-mediated immune resistance in the host intestinal tissue, while harmful pathogenic bacteria cause inflammatory intestinal injury. This eventually leads to the colonization and outbreak of invasive fungi in preterm infants.

## Data availability statement

The datasets presented in this study can be found in online repositories. The datasets have been submitted to NCBI (https://www.ncbi.nlm.nih.gov) with accession PRJNA821364 and PRJNA821375.

## Ethics statement

All the studies followed the guidelines for the ethical treatment of human specimens and were approved by the Ethics Committee of Guangzhou Women and Children's Medical Center. According to the Declaration of Helsinki, written informed consent was obtained from the parents of all enrolled neonates. Animals in this study were approved by Guangzhou Medical University's Institutional Animal Care and Use Committee and conducted in accordance with institutional guidelines.

## Author contributions

DH, HL and PW contributed to the study design. JL and CL joined in experiments. BH and PW performed and/or contributed critically to all experiments and analyzed the data. PW and YL recruited the study cohort and collected samples. YL and YK collated and counted the clinical data of volunteers. BH and DH wrote the manuscript. BH, YK and WZ put forward valuable amendments to the article. All the authors have read and approved the final manuscript.

## Funding

Guangzhou Science and Technology Plan Project (No. 202102080247 to Ping Wang, No. 201904010484 to Wei Zhou), Basic and Applied Basic Research Foundation of Guangdong Province (No. 2020A1515110702 to Bing Huang, No. 2022A1515012354 to Ping Wang), Outstanding Youth Development Scheme of Nanfang Hospital, Southern Medical University (2021J012 to Bing Huang), National Natural Science Foundation of China (82000518, 82170566 and 82222011 to Bing Huang, 82003471 to Yashu Kuang).

## Conflict of interest

The authors declare that the research was conducted in the absence of any commercial or financial relationships that could be construed as a potential conflict of interest.

## Publisher’s note

All claims expressed in this article are solely those of the authors and do not necessarily represent those of their affiliated organizations, or those of the publisher, the editors and the reviewers. Any product that may be evaluated in this article, or claim that may be made by its manufacturer, is not guaranteed or endorsed by the publisher.

## Supplementary material

Supplementary Figure 1 Evaluation of microbiome composition in newborns. Three indexes of alpha diversity are displayed: **(A)** the Chao1 index, **(B)** the Shannon index, and **(C)** the Simpson index. The results are shown as mean ± SEM. There were no statistical differences in the three indexes. **(D)** Principal component analysis (PCA): color matching of each sample in groups. The fraction of diversity captured by the coordinate is given as a percentage. Relative proportions of sequences read at the genus **(E)** levels assigned to different bacteria. The microbiome abundance was less than 0.5% in all samples, and the unannotated microbiomes were all merged into Others. Comparison of relative abundance of bacteria at genus **(F)** levels. The results are mean ± SEM, and the P values of the differences between groups were tested by the Mann-Whitney U test. *P < 0.05 vs Control, #P < 0.05 vs Control, +P < 0.05 vs Fungal. **(G)** A GraPhlAn was used to examine the overall microbiota composition at the phylum-to-genus level of all samples.
