# Supplementary figures and images for: Antibiotic-induced depletion of Clostridium species increases the risk of secondary fungal infections in preterm infants
Source: Front Cell Infect Microbiol. 2022 Aug 31;12:981823. doi: 10.3389/fcimb.2022.981823 (PMC9473543; doi:10.3389/fcimb.2022.981823)

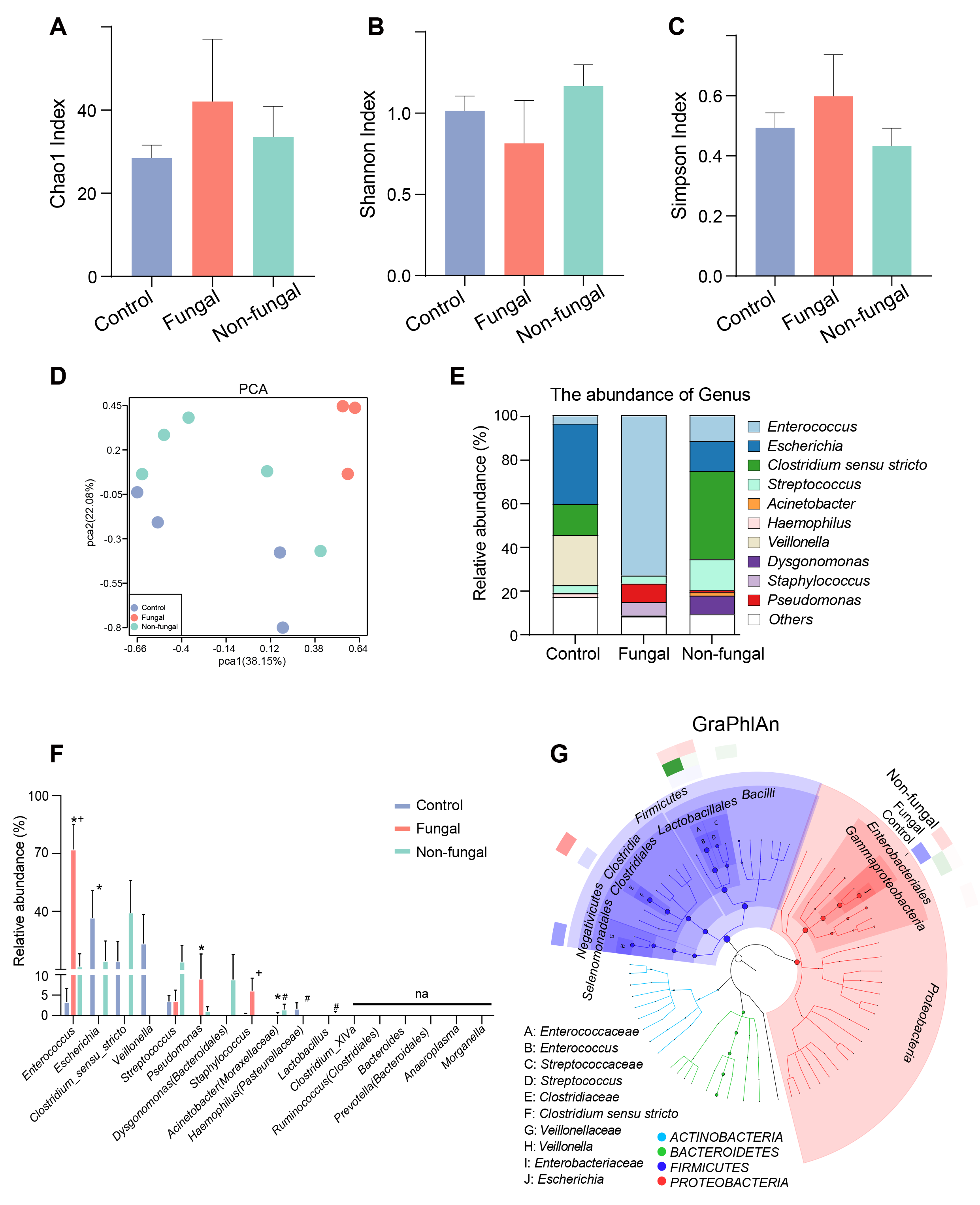

Supplement: Supplementary Figure 1 — Evaluation of microbiome composition in newborns. Three indexes of alpha diversity are displayed: (A) the Chao1 index, (B) the Shannon index, and (C) the Simpson index. The results are shown as mean ± SEM. There were no statistical differences in the three indexes. (D) Principal component analysis (PCA): color matching of each sample in groups. The fraction of diversity captured by the coordinate is given as a percentage. Relative proportions of sequences read at the genus (E) levels assigned to different bacteria. The microbiome abundance was less than 0.5% in all samples, and the unannotated microbiomes were all merged into Others. Comparison of relative abundance of bacteria at genus (F) levels. The results are mean ± SEM, and the P values of the differences between groups were tested by the Mann-Whitney U test. *P < 0.05 vs Control, #P < 0.05 vs Control, +P < 0.05 vs Fungal. (G) A GraPhlAn was used to examine the overall microbiota composition at the phylum-to-genus level of all samples. [file Image_1.tif]
